# Teaching an old ‘doc’ new tricks for algal biotechnology: Strategic filter use enables multi-scale fluorescent protein signal detection

**DOI:** 10.3389/fbioe.2022.979607

**Published:** 2022-09-23

**Authors:** Sergio Gutiérrez, Gordon B. Wellman, Kyle J. Lauersen

**Affiliations:** Bioengineering Program, Biological and Environmental Sciences and Engineering Division, King Abdullah University of Science and Technology (KAUST), Thuwal, Saudi Arabia

**Keywords:** fluorescent protein, microalgae, fluorescence, molecular tools, epitope tags

## Abstract

Fluorescent proteins (FPs) are powerful reporters with a broad range of applications in gene expression and subcellular localization. High-throughput screening is often required to identify individual transformed cell lines in organisms that favor non-homologous-end-joining integration of transgenes into genomes, like in the model green microalga *Chlamydomonas reinhardtii*. Strategic transgene design, including genetic fusion of transgenes to FPs, and strain domestication have aided engineering efforts in this host but have not removed the need for screening large numbers of transformants to identify those with robust transgene expression levels. FPs facilitate transformant screening by providing a visual signal indicating transgene expression. However, limited combinations of FPs have been described *in alga* and inherent background fluorescence from cell pigments can hinder FP detection efforts depending on available infrastructure. Here, an updated set of algal nuclear genome-domesticated plasmid parts for seven FPs and six epitope tags were generated and tested in *C. reinhardtii*. Strategic filter selection was found to enable detection of up to five independent FPs signals from cyan to far-red separately from inherent chlorophyll fluorescence in live algae at the agar plate-level and also in protein electrophoresis gels. This work presents technical advances for algal engineering that can assist reporter detection efforts in other photosynthetic host cells or organisms with inherent background fluorescence.

## Introduction

Since its discovery and conversion into a genetically coded reporter, the *Aequorea victoria* green fluorescent protein (*Av*GFP) has served numerous roles in molecular biology and medical research applications (Tsien, 1998). From this protein, cyan, green and yellow variants have been generated through mutagenesis, brightness and monomeric states engineered, and their use as genetically encoded reporters of gene expression established (Shaner et al., 2007). The *Av*GFP sequence led to the discovery of other similar proteins throughout the Cnidarian phylum. Red fluorescent variants were identified and optimized, other source organisms as well as even completely synthetic fluorescent reporters were developed from its sequence and structure (Verkhusha and Lukyanov, 2004). Proteins within the GFP-like class are identifiable by their β-barrel structure (Duwé and Dedecker, 2019). Non-fluorescent yet colored chromoproteins of corals sharing the β-barrel structure also find use as reporters (Liljeruhm et al., 2018). Fluorescent reporters have been used as tools across kingdoms, with their only use-caveat that they require molecular oxygen for the autocatalytic formation of their chromophore (Shaner et al., 2007). Fluorescent reporters enable differentiation, localization, quantification, and activation of molecular events. Technologies to visualize and monitor fluorescence have developed in stride with the advances in the design of proteins themselves; an investigator has options for fluorescence analysis in spectrophotometry, camera-capture, microscopy, and flow-cytometry.

The use of fluorescent proteins (FPs) as fusion-partners and indicators of transgene expression has been of particular value for genetic engineering in the eukaryotic model green microalga *Chlamydomonas reinhardtii* [Reviewed in (Lauersen, 2019)]. This organism is known to readily take up foreign DNA and integrate it into its nuclear genome through non-homologous end joining (NHEJ) (Kindle, 1990; Gumpel et al., 1994). A feature that has enabled forward genetic mutation studies of the nuclear genome of this alga through random integration of antibiotic selection markers and knock-out of native genes (Rochaix, 1995; Zhang et al., 2014; Li et al., 2019). The over-expression of transgenes from the nuclear genome of *C. reinhardtii*, however, requires sophisticated transgene design (Baier et al., 2018, 2020; Jaeger et al., 2019; Einhaus et al., 2021), strain domestication (Neupert et al., 2009, 2020; Dementyeva et al., 2021), and high-throughput screening of transformants mediated by FP fusions to target recombinant sequences (Abdallah et al., 2022) to identify high transgene-expressing cell lines which can arise from multiple integration events (Shahar et al., 2020) and ‘position effects’ of NHEJ integration (Kong et al., 2014).

Genetic fusion of target recombinant proteins with FPs has become a common strategy to identify high transgene expressing transformants in a population and facilitate genetic/metabolic engineering concepts with *C. reinhardtii* (Lauersen et al., 2016, 2018; Wichmann et al., 2018; Perozeni, 2020; Einhaus et al., 2021; Einhaus et al., 2022; Freudenberg et al., 2021; Freudenberg et al., 2022a). Target protein-FP fusions allow rapid identification of cell lines exhibiting robust transgene expression and translation as stronger fluorescence signal correlates to higher abundance of target-fusion protein products. Agar-plate level fluorescence imaging allows detection of microbial colonies that readily express target transgene-FP fusions against a large background of other clonal cell lines (Lauersen et al., 2015; Abdallah et al., 2022). Previous engineering in *C. reinhardtii* has employed a limited number of fluorescent cyan, yellow, and red emitting FPs using stock filter-sets to differentiate these signals from chlorophyll autofluorescence in stereo microscopy and in whole-plant imaging devices at the agar plate-level (Lauersen et al., 2015, 2016). However, this limited number of reporters prevents further gene stacking using FP-fusions as a screening technique and the filter selections were not optimized to maximize reporter detection.

Here, a broad set of algal-optimized Cnidarian and synthetic fluorescent reporters were expressed *in alga* and screened with curated narrow bandpass filters to extend the visual range of fluorescence detection to the edge of chlorophyll autofluorescence in *C. reinhardtii*. Several technical advances were achieved and are presented here to increase the number of reporters available for trait stacking in this alga. The techniques presented facilitate rapid screening of algal transformants at the agar plate level, the visualization of target-FP fusion molecular masses in protein electrophoresis gels, and introduce new species-specific molecular tools for the *C. reinhardtii* research community. These results are presented with specific application to *C. reinhardtii* engineering, however, may influence broader adoption of curated filter selection in analytic equipment design and influence reporter choices in other organisms with background fluorescence signals.

## Materials and methods

### *Chlamydomonas reinhardtii* culture conditions

*Chlamydomonas reinhardtii* strain UPN22 (Abdallah et al., 2022) was used for this work. UPN22 is a modified derivative of strain UVM4 (Neupert et al., 2009) a strain which was generated by Dr. Juliane Neupert in the lab of Prof. Dr. Ralph Bock that has improved nuclear transgene expression due to a Sir2-type histone deacetylase (SRTA) mutation (Neupert et al., 2020). UVM4 was provided to Prof. Kyle J. Lauersen (KJL) under material transfer agreement (MTA) between the Max Planck Institute of Molecular Physiology and KAUST and UPN22 was developed at KAUST as recently described (Abdallah et al., 2022). Briefly, the plastid genome of UPN22 has been modified with the pPO3 plasmid (Changko et al., 2020) to enable growth on phosphite and its nitrate metabolism was restored by a dual transformation of *nit1* (pMN24 (Fernández et al., 1989)) and *nit2* (pMN68 (Schnell and Lefebvre, 1993)) loci (Abdallah et al., 2022). UPN22 cultures were routinely maintained on agar-solidified TAPhi-NO_3_ medium (Abdallah et al., 2022) modified from Tris-acetate-phosphate (TAP) medium with phosphate replaced with phosphite (Gorman and Levine, 1965) and updated trace element solution (Kropat et al., 2011). Cultivation was performed at room temperature in shake flasks or microtiter plates containing liquid TAPhi-NO_3_ under 150 ¹mol photons m^−2^ s^−1^ mixed cold and warm LED lighting with 120-190 rpm agitation when required. Amido black (150 mg L^−1^) was added to TAPhi-NO_3_ agar by dissolving into the medium before autoclaving.

### Plasmid design, transformation, and mutant screening

**FIGURE 1:**
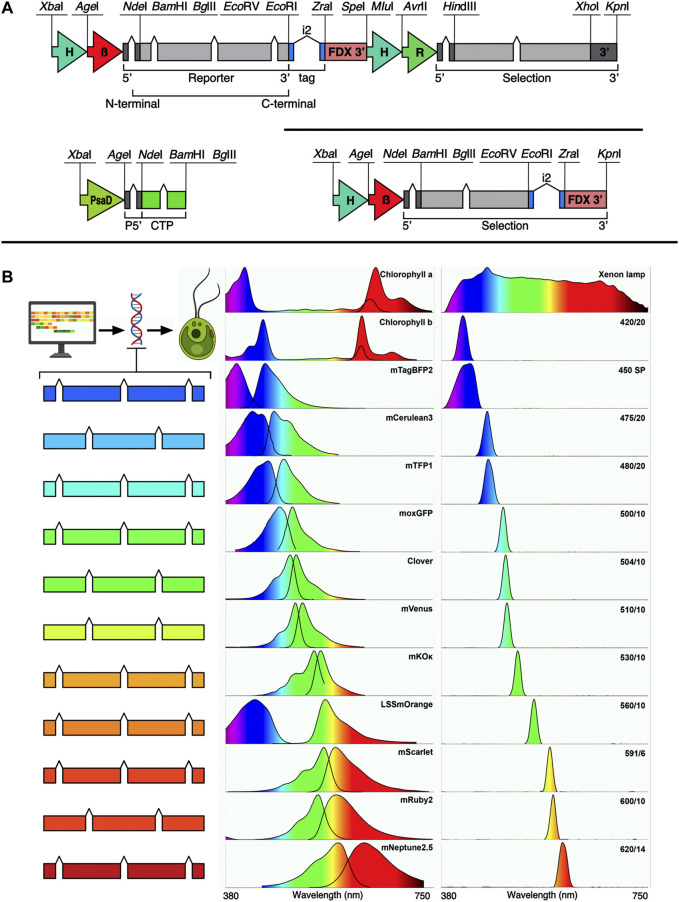
pOptimized 3.0 plasmid design, new fluorescent reporters, spectral properties and filters used in this work. **(A)** Design of the pOpt3 plasmids is shown: all intentional restriction enzymes are labelled, H-β—combined HSP70A beta tubulin promoter and its 5’ untranslated region (5’ UTR) (Einhaus et al., 2021). FDX 3’–3’ UTR of the ferredoxin 1 gene (Einhaus et al., 2021). Chevrons indicate placement of the RBCS2 intron 1, i2—indicates placement of the RBCS2 intron 2 inside coding regions for epitope tags. The selection marker cassette of the binary plasmid contains H-R—HSP70A-rbcs2 promoter and rbcs2 terminator (Wichmann et al., 2018). PsaD—photosystem I reaction center subunit II promoter with 5’ UTR containing and rbcs2i1 and its own chloroplast targeting peptide (CTP). Each element is separated by a unique restriction endonuclease site as indicated. **(B)** Left—fluorescent reporters optimized for *C. reinhardtii* nuclear transgene are shown in color coded cartoons representing the number of RBCSi1 each contains. Center panel—the excitation emission spectra for each corresponding FP and chlorophyll a + b (data from FPbase.org). Right panel—the relative transmittance (peak/bandpass nm) of each filter used in this work measured with a spectrometer. The light source used was a xenon lamp with broad emission spectrum which is shown in the top right.

The pOptimized (pOpt) plasmid set for transgene expression from the *C. reinhardtii* nuclear genome was first developed as described in (Lauersen et al., 2015) and was updated to its second iteration (pOpt2) with increased selection markers and reporters and a smaller nucleotide footprint in (Wichmann et al., 2018). In (Abdallah et al., 2022), the promoter and terminator of the pOpt2 plasmid gene of interest cassette as well as intron structure in the mVenus reporter was modified to match optimized designs from (Baier et al., 2020; Einhaus et al., 2021; Freudenberg et al., 2021). The plasmid and regulatory elements, coupled with algal-optimized synthetic transgenes (Jaeger et al., 2019) wherein systematic spreading of endogenous introns is used in codon optimized genes of interest, has been shown to enable reliable transgene expression in this host (Baier et al., 2018, Baier et al., 2020). Here, pOptimized3 (pOpt3) has been generated (Figure 1A), which was designed *in silico* and synthesized *de novo* to match requirements for both pOptimized and modular cloning (MoClo (Crozet et al., 2018)) standards by removing all potential interfering restriction sites from individual elements. The genetic elements built for this work include the *C. reinhardtii* photosystem I reaction center subunit II (PsaD) promoter and its chloroplast targeting peptide (CTP) with synthetic intron additions into the 5’UTR and CTP. A modified ferredoxin 1 (FDX1) 3’ terminator region was also used (Einhaus et al., 2021). The heat-shock70A beta-tubulin promoter from (Einhaus et al., 2021; Abdallah et al., 2022) was also included for cytoplasmic localization of transgenes. The pOpt2 concept introduced the minimal spectinomycin (MinS) expression cassette plasmids, which placed the *aadA1* gene in the GOI cassette without a second cassette, to enable direct fusion to the spectinomycin resistance gene of targets to be expressed (Wichmann et al., 2018). Here, we modified the MinS concept with the same promoter and terminator combinations of the pOpt3 GOI cassette. The genetic parts coding for reporters used in pOpt2 (mCerulean3, Clover, mVenus, and mRuby2 (Wichmann et al., 2018)) were recently redesigned to contain two copies of the ribulose bisphosphate carboxylase small subunit 2 (rbcs2) intron 1 (i1) (Abdallah et al., 2022). Here, new reporter genetic parts were made for other investigated reporters with three copies of rbcs2i1 to leverage the intron mediated enhancement effect of this element (Baier et al. 2018). Synthetic elements coding for C-terminal epitope tags were designed to include the intron 2 (i2) as its placement in this orientation has been shown to enhance transgene expression (Eichler-Stahlberg et al., 2009; Baier et al., 2018, 2020). All reporter amino acid sequences were taken from FPbase (www.fpbase.org), codon-optimized, and introns spread using the Intronserter algorithm (Jaeger et al., 2019).

After *in silico* design, all plasmids were synthesized *de novo* by Genscript. Plasmids were delivered as lyophilized DNA, transformed and maintained in *Escherichia coli* DH5a with ampicillin as a selection agent in lysogeny broth (LB). In addition, versions of each reporter were designed and synthesized for recombinant protein expression from the pET24 plasmid in *E. coli* (kanamycin resistance, BL21(DE3) cells). All plasmids have been deposited at the Chlamydomonas Resource Center (https://www.chlamycollection.org), their description and sequences are presented in Supplementary Material S1. Purified plasmids were prepared using a Qiagen plasmid mini prep kit on overnight 5 mL *E. coli* cultures following manufacturer’s instructions. Pre-algal transformation, 10 µg of each plasmid was linearized in 100 µL reactions with Thermo Scientific FastDigest restriction enzymes *Xba*I and *Kpn*I.

*C. reinhardtii* transformation was performed by glass bead agitation in the presence of 10 µg plasmid DNA as previously described (Kindle et al., 1989; Lauersen et al., 2015). Colonies were recovered after 1 week by plating on selective TAPhi-NO_3_ media with 15 μg mL^−1^ paromomycin as a selection agent. Upon recovery, colonies on primary transformant plates were picked using the Singer Instruments PIXL robot to TAPhi-NO_3_ plates, allowed to recover for ∼1 week and then stamped to fresh medium and plates containing amido black (150 mg L^−1^) using a Singer Instruments ROTOR robot. Amido black-containing plates were allowed to grow for 2-24 h before imaging.

### Fluorescence imaging using a gel documentation system

Iterative testing of different filters was implemented to select those with specific bandpass ranges that would allow selective excitation and emission of target fluorescent proteins *in alga* separate from chlorophyll (a or b) autofluorescence interference and to differentiate individual emission signals from one another (Figure 1B). Filters were tested in a ChemStudio Plus (Analytik Jena, United States) gel documentation system with an eLite xenon lamp and filter wheel extension (Analytik Jena, United States). The xenon lamp provides a broad-spectrum visible light emission (Figure 1B) and 8 individual mounted filter slots. The ChemStudio Plus has a built-in emission filter wheel for 5 × 50 × 50 mm filters in front of a 4-megapixel camera. Filters were sourced from either Omega Optical (United States) or Semrock (distributed by AVR Optics United States) as indicated in Table 1. Their bandpass transmission was captured using a Sekonic C-7000 SPECTROMASTER spectrophotometer (Figure 1B, data in Supplementary Material S2). Excitation filters were ground to fit mounts inside the ChemStudio PLUS eLite halogen lamp extension by the manufacturer (Analytik Jena, United States) or used as 50 × 50 mm pieces for the emission filters.

**TABLE 1:** Optical properties, use, and sources of filters tested in this work.

Product code	Company	Transmittance (nm)	Use in this work
Custom request	Omega optical	425/20	Excitation
Custom request	Omega optical	450SP	Excitation
Custom request	Omega optical	475/20	Excitation
Custom request	Omega optical	480/20	Emission
Custom request	Omega optical	500/10	Emission
Custom request	Omega optical	504/10	Excitation
Custom request	Omega optical	510/10	Emission
Custom request	Omega optical	530/10	Emission
Custom request	Omega optical	560/10	Excitation
FF01-591/6	AVR optics/Semrock	591/6	Excitation
Custom request	Omega optical	600/10	Emission
FF01-620/14	AVR optics/Semrock	620/14	Emission
DNA gel filter	Analytik jena	640/160	Emission

Fluorescence images of *C. reinhardtii* colonies on agarplates and protein gels were captured with different combinations of excitation and emission filters and exposure times to collect signals from each fluorophore (Figure 1B and Table 2) as listed in Supplementary Table S1. Long Stokes shifted (LSS) orange-red reporters were compared as listed in Supplementary Table S2 with the filter settings noted for LSSmOrange.

**TABLE 2:** Fluorescent proteins highlighted in this work.

Protein name	Excitation: Emission λ max	References
mTagBFP2	399:454	Subach et al., 2011
mCerulean3	433:475	Markwardt et al., 2011
mTFP1	462:492	Ai et al., 2006
moxGFP	486:510	Costantini et al., 2015
Clover	505:515	Lam et al., 2012
mVenus	515:527	Kremers et al., 2006
mKOκ	551:563	Tsutsui et al., 2008
LSSmOrange	437:572	Shcherbakova et al., 2012
mScarlet	569:594	Bindels et al., 2017
mRuby2	559:600	Lam et al., 2012
mNeptune2.5	599:643	Chu et al., 2014

### In-gel fluorescence and Western blotting

Expression of fluorescent proteins (mTagBFP2, mCerulean3, mTFP1, moxGFP, Clover, mVenus, mKOκ, LSSmOrange, mScarlet, mRuby2, mNeptune2.5) and epitope tags (StrepII: WSHPQFEK, HA: YPYDVPDYA, c-myc: EQKLISEEDL, E-tag: GAPVPYPDPLEPR, FLAG: DYKDDDDK, TAP: CSSGALDYDIPTTASENLYFQ, V5: GKPIPNPLLGLDST) in selected transformants was observed by a combination of sodium dodecyl sulfate–polyacrylamide gel electrophoresis (SDS-PAGE) in-gel fluorescence and Western blotting. Fluorescent proteins in gels were imaged with the same excitation/emission filter and exposure settings used for their *in alga* colony imaging. 504/10 nm excitation and no emission filter (open) were used to contrast and visualize the pre-stained protein marker (New England BioLabs, United Kingdom). Western blots were performed using polyvinylidene difluoride (PVDF) membranes and commercially available horseradish peroxidase (HRP)-linked monoclonal antibodies for each epitope tag (THE^TM^-tag products, Genscript).

Briefly, algae were cultivated in 12-well microtiter plates in TAPhi-NO_3_ until they reached the mid-log phase, 2 mL was centrifuged at 3,000x *g* for 2 min, the supernatant discarded, the pellet snap-frozen in liquid nitrogen, and stored at -80°C until further analysis. To lyse cells, samples were thawed on ice and the pellet resuspended in 200 μL of sample buffer without β-mercaptoethanol (0.2 M SDS, 0.3 M Tris, 30% glycerol, 0.02% bromophenol blue), vortexed, then incubated at 40°C for 5 min and placed on ice until loading on a gel. 20 µL of each sample was loaded into SDS-PA gels (5% stacking, 15% resolving) with 5 µL marker lane(s). After electrophoresis separation, gels were incubated for 20 min in ddH_2_O at room temperature and washed twice with water to remove excess SDS and allow refolding of fluorescent proteins. In-gel fluorescence was visualized in the ChemStudio PLUS using filter combinations for each fluorescent protein according to Supplementary Table S2.

For Western blotting, proteins were transferred to PVDF membranes by electrophoresis (50 V) at 4°C overnight in transfer buffer (0.02 mM Tris-Base, 0.2 mM glycine, 20% methanol). After transfer, membranes were blocked with blocking buffer (5% bovine serum albumin (BSA) (Fisher BioReagents, PA, United States), 5% milk powder (Almarai, Saudi Arabia) in Tris-buffered saline-Tween20 (TBS-T: 0.1% (v/v) polysorbate 20 in 0.1 mM tris-base, 0.1 M sodium chloride) for 1.5 h with gentle agitation at room temperature. After blocking, the membranes were rinsed three times for 5 min with TBS-T. For each antibody tested, a different SDS-PAGE was run with the same cell extract samples, membranes were individually incubated with different conjugated antibodies for each respective tag for 1 h with gentle agitation (50 rpm). All antibodies used are from GenScript: THE^TM^ HA-tag antibody (1:500), THE^TM^ c-myc-tag antibody (1:1000), THE^TM^ E-tag antibody (1:1000), THE^TM^ FLAG-tag antibody (1:2000), THE^TM^ TAP-tag antibody (1:2000), THE^TM^ V5-tag antibody (1:1000), THE^TM^ GFP-tag antibody (1:5000), THE^TM^ NWSHPQFEK-tag antibody (StrepII) (1:3000). After incubation, the membranes were washed three times for 10 min with TBS-T. Finally, each membrane was incubated with a mixture of the chemiluminescent substrates (Bio-Rad laboratories, United States) at a 1:1 ratio for visualization. Luminescence signals were captured in the ChemStudio PLUS camera with no emission filter for 2 min or as needed to visualize signals.

### Flow cytometry of fluorescent *C. reinhardtii* transformants

Liquid cultures were analyzed by flow cytometry using an Invitrogen Attune NxT flow cytometer (Thermo Fisher Scientific, United Kingdom) equipped with a CytKick microtiter plate autosampler unit using a 488 nm blue laser for forward scatter (FSC) and side scatter (SSC): the machine is equipped with 405 nm (Violet, VL), 488 nm (Blue, BL), 561 nm (Yellow, YL), and 638 nm (Red, RL) excitation lasers and corresponding emission filter sets provided by the manufacturer. Transformants were grown in a 12-well plate as described for protein harvest until they reached the mid-log phase, then diluted 1:1000 with 0.9% (w/v) NaCl solution and 200 μl of each diluted sample was loaded into a 96-well microtiter plate placed in the autosampler. Samples were mixed five times immediately before analysis, and the first 25 μL of the sample was discarded to ensure a stable cell flow rate during measurement. Data acquisition stopped when 50 μl from each well was analyzed, with technical triplicates (*n* = 3). For dual population sampling, 100 μL of diluted transformant and 100 μL diluted wild-type were mixed and measured as above. The Attune NxT Software v3.2.1 (Life Technologies, United States) was used to perform all post-acquisition analysis and population clustering.

## Results and discussion

For the model microalga *C. reinhardtii*, the emission signals from FPs have been leveraged to identify individual cell lines in a population of transformed and selected colonies that robustly express a target transgene-FP fusion of interest (Mackinder et al., 2016, 2017; Lauersen, 2019). Previous reports have demonstrated that multiple fluorescent proteins can be combined in the alga cell simultaneously by transformation and mating using micro-plate readers and confocal microscopy imaging techniques (Rasala et al., 2013, 2014). FP expression can also screened at the agar-plate level with whole-plant or stereo-microscopy imaging apparatus, a technique which assists selection of robust expressing cell lines quickly from a population (Lauersen et al., 2016, 2018; Wichmann et al., 2018; Einhaus et al., 2021; Freudenberg et al., 2021, 2022a, 2022b). Fluorescent proteins are not the only bio-molecules which emit some amount of fluorescence signal in host cells and tissues. In Chlorophyceae, chlorophyll a+b emit fluorescence strongly in red wavelengths of visible light (Figure 1B), as does phycocyanin in cyanobacteria and red algae. This autofluorescence of biomolecules can hinder detection of target fluorescent events if technical infrastructure is not optimized to enable narrow bandpass visualization of light wavelengths.

Reports of metabolic engineering from nuclear genome transgene expression in the alga *C. reinhardtii* have combined up to three separate FPs (cyan, yellow, and red) fused to target enzymes expressed in the same strain (Lauersen, 2019). Screening independent signals separate from chlorophyll autofluorescence was possible due to spectral distance between excitation and emission of the mCerulean3 (cyan), mVenus (yellow), and mRuby2 (red) FPs and chlorophylls. However, two issues were apparent in these efforts: first, mCerulean3 was challenging to visualize *in alga* unless it was highly expressed; second, having only 3 FP colors limited the multiplexing of fusion partner transgene targets which could be combined in one algal cell line.

**FIGURE 2:**
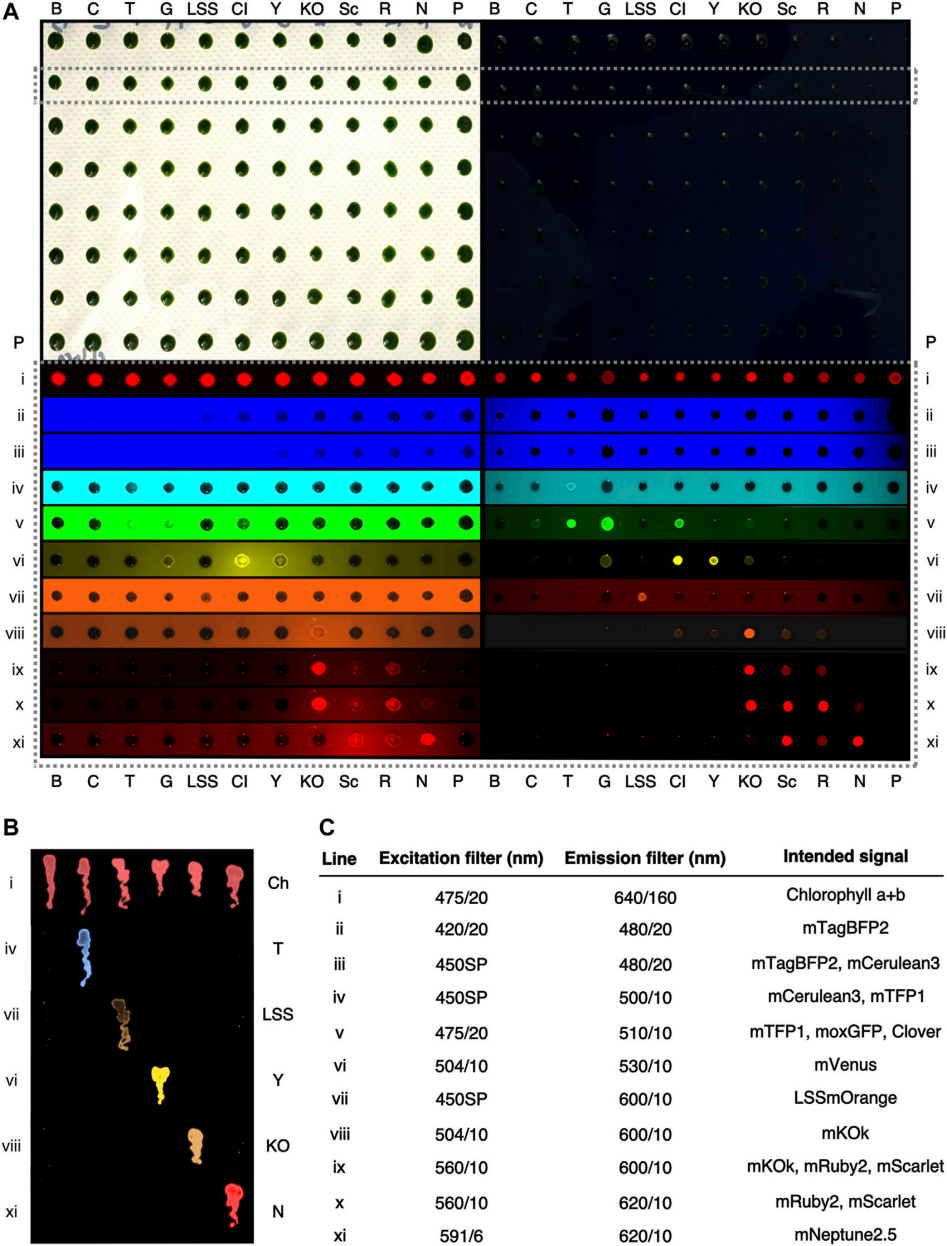
Representative fluorescence behaviors *C. reinhardtii* colonies expressing different fluorophores at the agar plate level. **(A)**—A digital camera color image of two 96-colony orientation agar plates of *C. reinhardtii* cells. Plates are stamped replicas of each other, with the right plate containing 150 mg L^−1^ amido black in the medium. **(B)** mTagBFP2, **(C)**—mCerulean3, T—mTFP1, G—moxGFP, LSS—LSSmOrange, Cl—Clover, Y—mVenus, KO—mKOk, Sc—mScarlet, R—mRuby2, N, mNeptune2.5, P—parent strain. A single row (dashed box) is highlighted and signals from this row shown after excitation and emission imaging with the indicated filters (i-xi). Colors have been digitally added to greyscale images by the VisionWorks software. Both plates were imaged simultaneously with the indicated filters. **(B)** An amido black plate with streaks of *C. reinhardtii* colonies expressing individual fluorescent proteins and imaged with the indicated filters colour has been digitally added to greyscale images. Ch—chlorophyll a+b autofluorescence. **(C)** filters used in **(A,B)** and their intended fluorescent protein visualization.

The goal of this work was to identify other FPs and filters which could be combined to allow increased independent fluorescence signal detection *in alga* for improved transgene-FP loading in the photosynthetic cells. A broad range of fluorescent reporters were optimized for expression from the algal nuclear genome and a range of bandpass filters trialed to enable precise detection of different FP-expressing algae at the agar plate level (Figure 1). This curation enabled *in vivo* visualization of FPs in the photosynthetic cells across the visible spectrum, from blue to far-red, without interference from chlorophyll a+b autofluorescence. Separation of up to five independent FP reporters without spectral overlap was achieved, expanding the possibilities of multiplexing these signals for transgene expression combinations (Figures 1B, 2A, Supplementary Figure S1).

### Bandpass filters enable macro-fluorescence analysis across the visible spectrum

Independent transformant colonies of *C. reinhardtii* generated with plasmids for expression of eleven different fluorophores from the nuclear genome were generated by glass bead transformation and selection. Colonies generated from each plasmid were picked and screened with filters chosen relative to bandpass ranges in a gel-documentation (‘doc’) system equipped with multiple filter mounts as described in the methods (Figure 2, Supplementary Figure S1). Representative colonies expressing each fluorophore were then grouped and combined on a single selective plate in 96 colony orientation and replicated prior to imaging to compare fluorescence behaviors across fluorophores (Figure 2A).

Visualization of FP expression in the lower visible wavelength ranges (blue to green) was hindered by background fluorescence from agar-medium, which limited the visibility of fluorescence signals and individual colonies (Figure 2Aii-v, Supplementary Figure S1). This issue was less apparent in yellow-red wavelengths (Figure 2Avi-xi) which could be detected against agar autofluorescence. In order to reduce this interference, a strategy of medium dyeing was employed. Previously, amido black staining of agar has been used to reduce this background fluorescence (Wichmann et al., 2018), this technique was found here to improve signal-to-noise ratios and results in clearer identification of fluorescent colonies across the visible fluorescence spectrum (Figure 2A, lower right panel).

### Reporters in blue-cyan wavelengths

Given the aforementioned issues of mCerulean3 detection in alga, comparison of blue, cyan and green light-emitting FPs in plate-level analysis was conducted to identify a suitable replacement reporter. Although it was possible to detect mTagBFP2 in colonies at the agar plate level, even with amido black, it was difficult to distinguish this reporter from the background noise of the agar plate (Figure 2Aii). As expected, mCerulean3 was difficult to visualize on the agar plate, distinguishable only because these colonies had less-pronounced contrast than non-expressing colonies (Figure 2Aiii). In the cyan filter set, mTFP1 exhibited robust emission, clearly detectible (Figure 2Aiv). The emission of mTFP1 bled into green filter sets, being visible in filters with moxGFP and Clover (Figure 2Av); the emissions of these latter two also bled into the yellow ranges where mVenus was detected (Figure 2Avi). mTFP1 and mVenus, however, did not exhibit any spectral overlap and could be readily distinguished (Figures 2Aiv,vi, 2B). Due to separation from yellow FPs and its inherent brightness, mTFP1 is a valuable cyan replacement for mCerulean3.

### Long Stokes shifted reporters

We were interested in the application of long Stokes shifted (LSS) reporters as a means to add further independently detectible FPs in the algal cell. These reporters are stimulated with blue light and emit in the orange-red wavelengths (Shcherbakova et al., 2012). LSSmOrange, LSSmKate2, and LSSmCherry1 were compared by expression from the algal nuclear genome and agar-plate level screening (Supplementary Figure S2). Of these three reporters, LSSmOrange exhibited the most readily detectible signal on amido black plates when 450SP and 600/10 nm filters were combined (Supplementary Figure S2). Therefore, it was chosen for further use as a reporter in alga which can be independently visualized in combination with others without spectral overlap (Figure 2Avii).

### Reporters in yellow-red wavelengths

The mVenus (yellow) and mRuby2 (red) reporters have been widely used for *C. reinhardtii* transgene expression analysis (Lauersen, 2019) as these can be visualized with 504/10-530/10 and 560/10-600/10 excitation and emission filters on agar plates without amido black (Figure 2Avi). To further expand available reporters within the yellow-red wavelengths for the alga, orange (mKOk) and red (mRuby2, mScarlet) emitting FPs and respective bandpass filters were investigated here. Using 504/10 excitation and 530/10 emission, mVenus (and Clover) exhibited intense brightness, while mKOk does have some bleed through signal depending on exposure time (Figure 2Avi). mKOk is a very bright and valuable reporter in the orange-red wavelengths (Tsutsui et al., 2008), however, its signal caused significant bleed-through and overlap in the red channels. We found that, excitation with 560/10 filter yielded a robust signal in mKOk, but also from mRuby2 and mScarlet (Figure 2ix). It was, however, possible to distinguish mKOk from mRuby2 and mScarlet reporters by excitation with 504/10 and 600/10 nm emission filters (Figure 2Aviii). Therefore, it is possible to distinguish mVenus and mKOk emissions from one another at the agar plate level.

### Far-red separation from chlorophyll autofluorescence

Chlorophyll a+b emit fluorescence in red and infrared wavelengths; these signals can be used to help orient cells (algae and plants) during microscopy; however, the signal may also technically limit the detection of other reporters. Mixed chlorophyll emission signal of colonies on plates could be readily detected with 475/10 nm excitation (blue), a standard DNA-gel orange emission filter (transmittance from 560-720 nm) (Figure 2Ai). Chlorophyll b has a lower emission peak (645 nm) compared to chlorophyll a (671 nm) and both spectra begin emitting light at ∼625 nm. There exists a possibility of detecting a far-red signal before the emission wavelengths of chlorophyll.

The mNeptune2.5 reporter is a far-red shifted fluorescent protein with emission starting at 600 nm and reaching a maximum of 643 nm (Chu et al., 2014). Here, a 620/14 nm bandpass filter was found to not allow the fluorescence signal from either chlorophyll species to pass, however, detection of mNeptune2.5 fluorescence emission was observed (Figure 1B, Figure 2Axi, Supplementary Figure S1). The mNeptune2.5 reporter can also be separated from mKOk (orange) signals by selective use of a 591/6 nm excitation filter (Figure 2Axi). With this selective filter set, mNeptune2.5 is a new reporter that is able to be visualized in cells containing chlorophyll a+b (Figures 2Axi,Bxi). However, Cyanidiales algae which contain the protein photopigment phycocyanin (Lang et al., 2020), exhibited bleed-over of emission that prevents the use of this far-red reporter and filter combination, which would likely also be the case for cyanobacteria (Supplementary Figure S3).

### Choice of reporters across the spectrum

Given their distinct spectral properties, mTFP1, mVenus, LSSmOrange, mKOk, and mNeptune2.5 can be separately imaged using bandpass filters in *C. reinhardtii* cells. These reporters can be distinguished from one another in monochromatic camera imaging techniques using specific filters (Figure 2B, Supplementary Figures S1, S4 and Supplementary Table S2). Alternatively, mTagBFP2 and the green fluorescent reporters can substitute mTFP1 and mVenus, depending on analysis needs (Supplementary Table S2). Depending on gene expression, some spectral overlap in emission signal may occur, yet selective filter choice and exposure times can help differentiate one signal from another.

Direct target protein-FP genetic expression may be the most robust method for rapid determination of gene expression in alga. Of importance in future studies will be application of fluorescent signals from algal cells in further applications beyond target protein-FP fusions for other applications of engineered algal cells. Biosensors which transcriptionally respond to stimulation can use FPs as expression read-outs and consistent expression coupled to intensiometric fluorescence signals may also be developed similar to calcium sensors (Nasu et al., 2021). Future efforts of metabolic engineering in *C. reinhardtii* may benefit from chemical fluorescent probes which react to certain metabolite accumulation and could also be used in direct plate level fluorescence detection (Dai et al., 2021). Filter combinations for imaging each reporter tested here are stated in Figure 2C. The combination of reporters and filter sets here provides a rapid means of selecting and analyzing transgene expression in photosynthetic cells**,** although exposure times will vary based on available infrastructure.

### Selective filter sets allow *in vitro* protein visualization in gels

**FIGURE 3:**
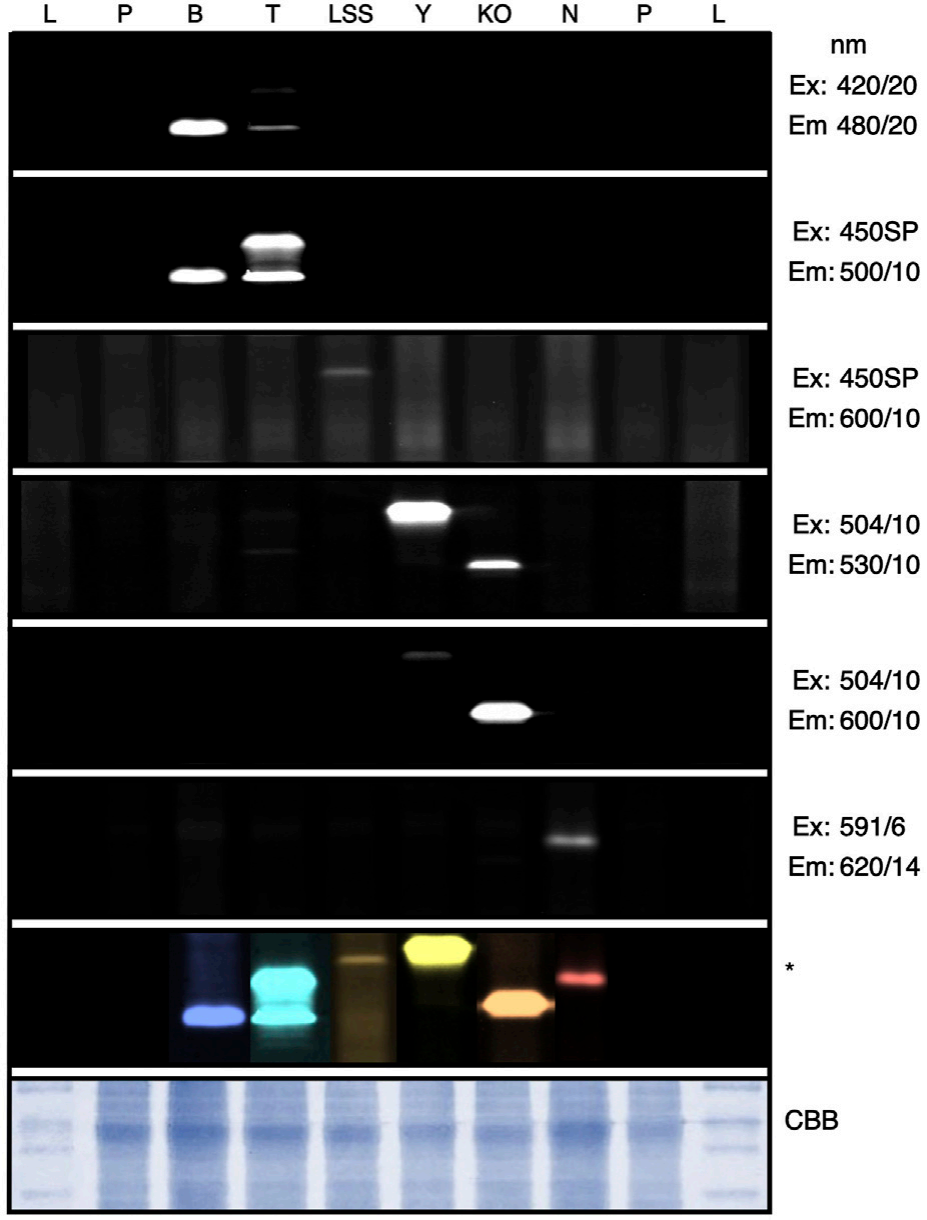
In-gel fluorescence screening of reporter protein signals. *C. reinhardtii* transformants expressing different fluorophores were subjected to SDS PAGE and imaged within the ChemStudio PLUS using different excitation (Ex) and emission (Em) filter sets as indicated. Individual signals from each reporter can be seen as bands, which were compiled into a composite image and artificially colored by Affinity photo (Serif, EU). CBB—Coomassie brilliant blue staining of the protein gel. L—Protein ladder, P—Parent strain, B—mTagBFP2, T—mTFP1, LSS—LSSmOrange, Y—mVenus, KO—mKOk, N—mNeptune2.5. Full gel images can be found in Supplementary Figure S6.

Currently, Western blotting is a standard for target protein expression quantification and to confirm apparent protein molecular mass after gel electrophoresis. The curated filter sets used in colony screening above also enabled visualization of fluorescent proteins in gels when β-mercaptoethanol is left out of sample buffers (Figure 3, Supplementary Figure S5). Oligomerization and aberrant running behaviors were observed even in reporters with highly similar predicted molecular masses (Figure 3): mTagBFP2 and mTPF1 have expected molecular masses only 0.2 kDa in difference, yet mTPF1 exhibited dimer formation, mKOk should be the smallest protein at 24.5 kDa, yet its running behavior is similar to mTagBFP2, and mVenus (26.9 kDa) exhibited a running behavior in this gel higher than mNeptune2.5 (27.5 kDa). In-gel fluorescent protein detection can be of value if a non-precise molecular mass determination, yet confirmation of difference in size from control is needed.

The ability to use fluorescence for plate-level rapid colony screening and target recombinant protein visualization in gel allowed determination of the expression of two new transgene targets after integration into the algal nuclear genome: the non-ribosomal peptide synthase (NRPS) A from *Streptomyces lavendulae* (BpsA, NCBI: AB240063.1, (Hui et al., 2021)) and the 4′-phosphopantetheinyl transferase (4’-PPTase) from *Pseudomonas aeruginosa* (*Pa*PcpS, Protein Data Bank ID: 7BCZ_A, (Carivenc et al., 2021)). BpsA expression was chosen for several reasons: it is the first example of heterologous NRPS expression in a eukaryotic algal host, it is a validation of C-terminal fusion to mVenus using the current iteration of intron spreading, and the fusion protein is ∼170 kDa making it one of the larger heterologous proteins to be expressed from the nuclear genome of this alga. The *Pa*PcpS acts as an activator for the BpsA and so we chose to express it as well, as a preliminary effort to plan transgene combinations. Both protein-coding sequences were codon-optimized for *C. reinhardtii* expression (Jaeger et al., 2019), synthesized and cloned 3’ of the mVenus reporter in pOpt3_mVenus plasmids (Supplementary Figure S6). Transgene expression was first screened at the agar plate level for YFP signal using 504/10-530/10 filter sets, then selected colonies with robust FPs expression were chosen and protein samples were run on SDS-PAGE followed by visualization using the same filter sets (Supplementary Figure S6). Both BpsA and *Pa*PcpS-YFP fusion proteins exhibited shifts in molecular mass as expected based on their predicted molecular masses indicating the value of in-gel fluorescence for molecular mass approximation (Supplementary Figure S6).

### A suite of optimized epitope tags complements fluorescence detection

**FIGURE 4:**
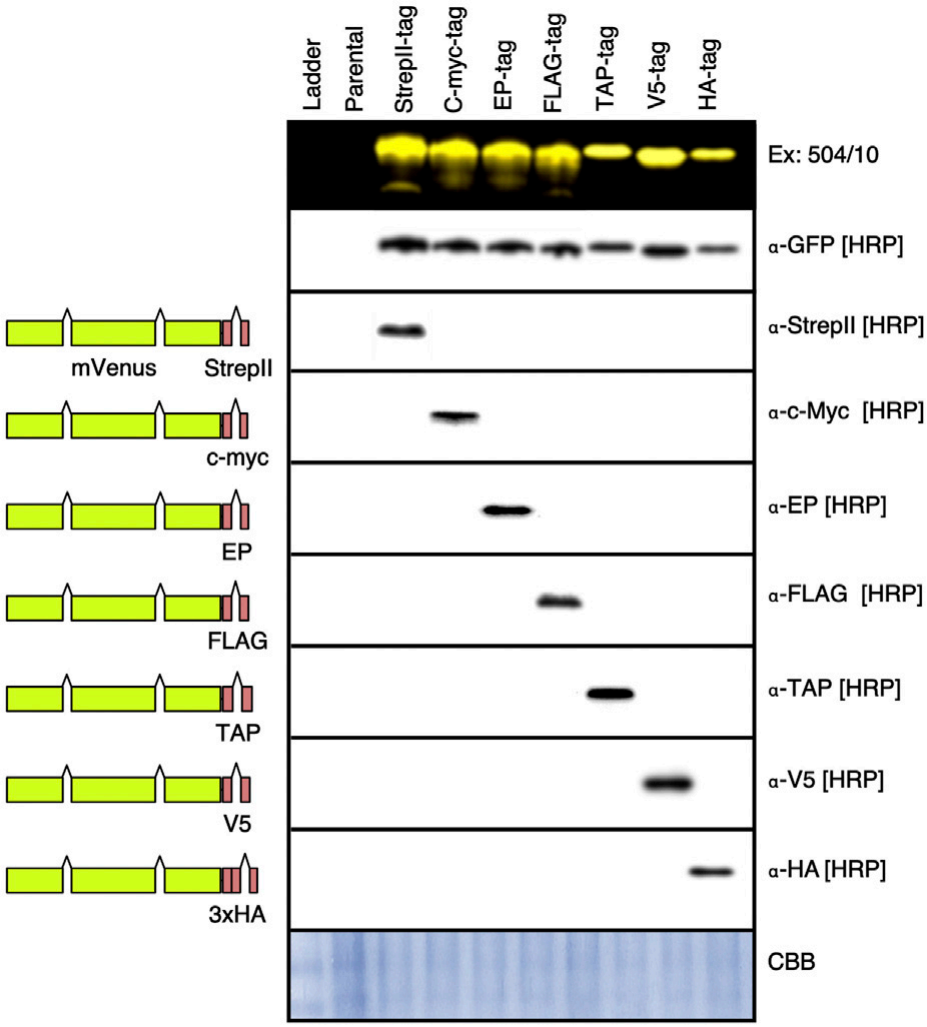
Western blot of different epitope tags expressed in *C. reinhardtii*. Plasmids were generated wherein the *Eco*RI-*Zra*I bordered fragment which contains a coding region for the StrepII epitope tag and rbcs2i2 were replaced by coding regions for the c-myc, E epitope (EP), FLAG, 21 amino acids of the tandem affinity purification (TAP), small epitope of the paramyxovirus 5 (V5), and 3-copy Human influenza hemagglutinin (3xHA) tags. Colonies expressing each mVenus-tag construct were run on 8 separate SDS PAGE gels, one visualized with 504/10-530/10 filters, then each were blotted onto PVDF membranes. Detection of each epitope was performed with the indicated antibodies as described in the methods section. Full blot images can be found in Supplementary Figure S7. HRP—horseradish peroxidase linked primary antibody.

In addition to fluorescence capacities, the ability to perform precise protein accumulation studies against internal standards relies on epitope tags and their respective antibodies. Here, seven different epitope tag genetic elements were constructed to fit with the updated pOpt3-parts and their capacity for low-background independent detection in Western blotting was tested (Figure 4, Supplementary Figure S7). Previously, the StrepII-tag has been reliably used in *C. reinhardtii* bioengineering studies as it has low background signal with algal cellular extracts. In this work, c-myc, EP, FLAG, TAP, v5, and 3xHA tags were all detectible independently without crossover using primary HRP-linked commercial antibodies when tags were fused to the C-terminus of the mVenus reporter expressed from the algal nuclear genome (Figure 4). In combination with in-gel fluorescence of the fluorescent reporters developed in this work, these optimized tags can also be used to increase specificity of molecular characterization of recombinant target expression.

### Advantages of camera imaging over other fluorescence analysis technologies

**FIGURE 5:**
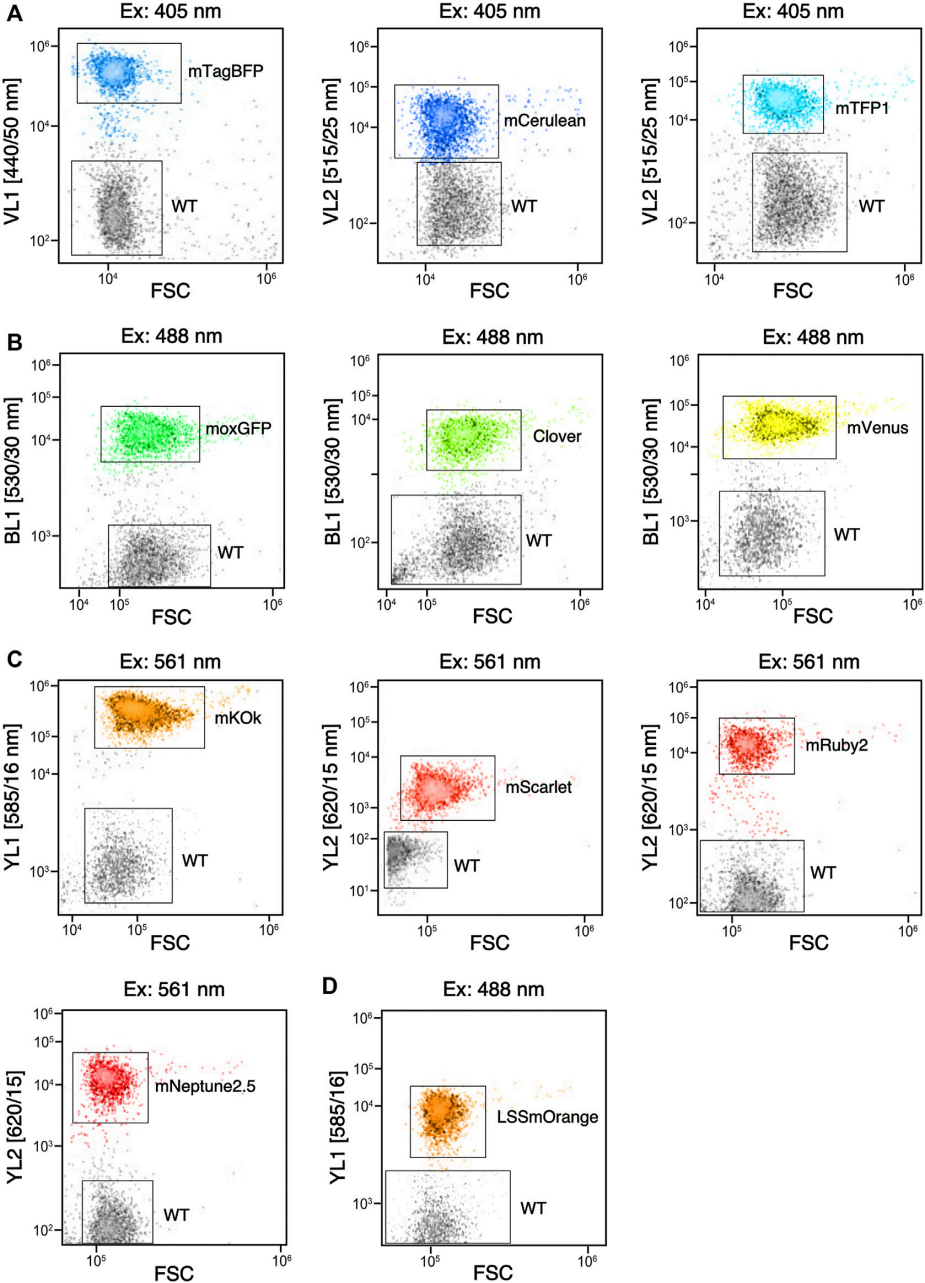
Flow cytometry of fluorophores expressed in *C. reinhardtii* that can be separated from wild type (WT). **(A)** signals obtained for three reporters compared to WT with violet laser (405 nm) excitation and emission against forward scatter (FSC) plot from respective filters (VL#). **(B)** signals obtained from three reporters compared to WT with blue laser (488 nm) excitation and emission from the BL1 filter plotted against FSC. **(C)** Signals obtained from 4 reporters compared to WT with orange laser (561 nm) excitation and emission from various filters (YL#) plotted against FSC. **(D)** Signal of LSSmOrange compared to WT when stimulated with 488 nm laser and visualized with YL1 emission plotted against FSC.

All FPs expressed in the algal cell were found to be readily visible in other fluorescence analysis methods like flow cytometry (Figure 5) or liquid plate readers (not shown). In flow cytometry fluorescence analysis, clear population distinction for each reporter based on excitation laser and emission filter combinations was observed (Figure 5). Flow cytometry techniques can provide significant data on cell densities, sizes, and expression intensity, all of which are valuable to understanding the dynamics of a transformed cell line. This technology is even more powerful when coupled with an automated sampling instrument. The need for mixing and resuspension in liquid cultures can make the analysis of a 384 population of cell lines last from 3.7-7.7 h (not shown), whereas camera-based imaging at the plate level can provide the advantage of a snapshot of reporter expression of a population in a matter of minutes (Supplementary Figure S6). With the added benefit of in-gel fluorescence presented above, adapting camera-capture technology with curated filter sets can be a valuable addition to FP analysis capabilities.

## Conclusion

In this work, a curated selection of narrow bandpass filters is shown to be a highly valuable technical addition to population screening and transgene expression analysis in the green alga *C. reinhardtii*. Selective filters were coupled to expanded algal genetic parts spanning the entire range of the visible color spectrum and precise filter selection was used to push the detection range of reporter emission to the edge of chlorophyll auto-fluorescence signals in the far-red. This work demonstrates that appropriate filter choice can enable fluorescent reporter detection in gels as well as *in vivo*, presenting multiple technical advantages to the user. A major limitation remains in use of blue emitting reporters for agar-plate level screening, the emission signal in these ranges is difficult to separate from agar fluorescence. However, these reporters are nevertheless detectible over parental strains in more precise detection technologies. Future efforts will be able to use the identified FPs and filter settings to stack higher numbers of transgenes in this alga and expand heterologous metabolic pathway engineering applications using these tools. The techniques presented in this work will likely be valuable to other non-model organisms and those where agar-plate level reporter expression screening can facilitate rapid identification of desired transformants from a population. It will also be of value in the development of other types of fluorescent reporters and their rapid detection in imaging systems. The filters presented here and their application in gel documentation, or ‘doc’, systems may encourage more optimized designs of these imaging devices to have broader capacities for fluorescence analysis.

## Data Availability

The original contributions presented in the study are included in the article/Supplementary Materials, further inquiries can be directed to the corresponding author.

## Supplementary material

SUPPLEMENTARY FIGURE S1 Artificial colored whole-plate images captured during fluorescence screening under different filter sets in the Analytik Jena ChestudioPLUS for colonies of Figure 2A. filter sets as indicate per image. **(H)**—digital color camera image of agar and amido black containing plates. **(B)**—mTagBFP2, **(C)**—mCerulean3, T—mTFP1, **(G)**—moxGFP, LSS—LSSmOrange, Cl—Clover, Y—mVenus, KO—mKOk, Sc—mScarlet, R—mRuby2, N, mNeptune2.5, P—Parent strain (furthest right column as well as top and bottom row on the plate). 6 colonies of each reporter were organized in columns in the middle of the plate.

SUPPLEMENTARY FIGURE S2 Colony fluorescence of three long Stokes shifted reporters on an amido black containing agar plate captured in the Analytik Jena ChemstudioPLUS. Artificial orange color on black and white image added in the VisionWorks software. Left—plasmid map cartoons of the expression cassette for the three reporters. Right—fluorescence signals of colonies. Filters and exposure time indicated above.

SUPPLEMENTARY FIGURE S3
*Cyanidioschyzon merolae* and *Galdieria sulphuriana* streaked onto (2x) modified Allen’s medium (MA2, (Minoda et al., 2004)) pH 1.95, 1% gellan gum plates, with or without 150 mg L^−1^ amido black, imaged with the indicated filter sets and exposure times.

SUPPLEMENTARY FIGURE S4 Raw images of whole-plate fluorescence screening under different filter sets for the streaked colonies in Figure 2B. *C. reinhardtii* cells expressing different fluorophores were streaked by hand onto an agar plate containing amido black and imaged after 24-h growth. Black and white images captured in the ChemStudio plus with indicated filters. Labels: P—Parent strain, T—mTFP1, LSS—LSSmOrange, Y—mVenus, KO—mKOk, N, mNeptune2.5.

SUPPLEMENTARY FIGURE S5 Raw images of in-gel fluorescence images captured using different filter sets. Filters and exposure times as indicated in figure.

SUPPLEMENTARY FIGURE S6 Cartoon representation of the mVenus-BpsA and mVenus-*Pa*PcpS expression plasmids, colony (right) and in-gel fluorescence expression analysis (left). Both fluorescence images were taken using 504/10 excitation and 530/10 emission filters for 30 s of exposure. Full plasmid sequences with annotations can be found in Supplementary Material S1. M—protein ladder (marker), lanes 1-3 transformants expressing mVenus-BpsA, 4-6 transformants expressing mVenus-*Pa*PcpS (PPTase), P—parent, C—empty control lane, YFP—transformant expressing the mVenus (YFP) reporter alone.

SUPPLEMENTARY FIGURE S7 Images of whole membrane exposures for Western blots of various epitope tags presented in Figure 4. —Antibodies as indicated per each blot image. **(I)**—CBB—Coomassie brilliant blue staining of one representative gel of 8. **(J)**—Fluorescence signal of mVenus reporter with the capture settings indicated for one representative gel also used in the blot of **(H)**.
