# Supplementary material for: Teaching an old ‘doc’ new tricks for algal biotechnology: Strategic filter use enables multi-scale fluorescent protein signal detection
Source: Front Bioeng Biotechnol. 2022 Sep 23;10:979607. doi: 10.3389/fbioe.2022.979607 (PMC9540369; doi:10.3389/fbioe.2022.979607)
Supplement: Supplementary file 1 [file Table1.DOCX]

**Supplemental Table 1.** Capture settings for fluorophores in Analytik Jena Chemstudio plus with eLite illumination.

| **Reporter** | **Excitation filter** | **Emission filter** | **Exposure (m:s)** | **Amido black?** |
| --- | --- | --- | --- | --- |
| mTagBFP2^†^ | 420/20, 440SP,450SP | 480/20 | 2:30 | Yes |
| mCerulean3^†^ | 450SP | 480/20, 500/10 | 2:30 | Yes |
| mTFP1^†^ | 450 SP, 475/20 | 500/10, 510/10 | 2:30, 0:30 | Yes |
| moxGFP | 475/20 | 510/10 | 0:30 | Yes |
| Clover | 475/20 | 510/10 | 0:30 | Yes |
| mVenus | 504/10 | 530/10 | 0:30 | No |
| mKOk | 504/10, 560/10 | 600/10 | 0:30 | No |
| LSSmOrange | 450SP | 600/10 | 1:30 | Yes |
| mScarlet | 560/10, 591/6^‡^ | 600/10, 620/14^‡^ | 0:30 | No* |
| mRuby2 | 560/10, 591/6^‡^ | 600/10, 620/14^‡^ | 0:30 | No* |
| mNeptune2.5 | 591/6, 600/10^◊^ | 620/14, 620/14^◊^ | 1:30 | No* |

^†^The combination of 420/20 excitation with 480/20 emission filter stimulates mTagBFP2 more than mCerulean3 or mTFP1, but not enough to distinguish them from one another.

*Although RFP variants can be detected without amido black plates, signal is clearer when amido black is used.

^‡^Use of 591/6 excitation and 620/14 emission filters allow separation of red proteins from mKOk. mNeptune2.5 is also visible in this combination

^◊^Separation can be achieved with the 600/10 excitation and 620/14 emission filter combination of mNeptune2.5 and other red fluorescence proteins, but some bleed through from mScarlet and mRuby2 does occur and is not recommended as a combination.

SP – short pass filter (allows photons below the indicated wavelength to pass).

**Supplemental Table 2.** Combinations of fluorophores that can be imaged simultaneously in samples with separate filter settings.

**Fluorophore combinations that can be separated simultaneously 1**

| Reporter | Excitation filter | Emission filter | Exposure (m:s) | Amido black? |
| --- | --- | --- | --- | --- |
| mTFP1^†^ | 450 SP, 475/20 | 500/10, 510/10 | 2:30, 0:30 | Yes |
| mVenus | 504/10 | 530/10 | 0:30 | No |
| LSSmOrange | 450SP | 600/10 | 1:30 | Yes |
| mKOk | 504/10 | 600/10 | 0:30 | No* |
| mNeptune2.5 | 591/6 | 620/14 | 1:30 | No* |

**Fluorophore combinations that can be separated simultaneously 2**

| Reporter | Excitation filter | Emission filter | Exposure (m:s) | Amido black? |
| --- | --- | --- | --- | --- |
| mTagBFP2 | 420/20, 440SP,450SP | 480/20 | 2:30 | Yes |
| moxGFP/Clover | 475/20 | 510/10 | 0:30 | Yes |
| LSSmOrange | 450SP | 600/10 | 1:30 | Yes |
| mKOk | 504/10 | 600/10 | 0:30 | No |
| mNeptune2.5 | 591/16 | 620/14 | 1:30 | No* |

*Amido black is still recommended to reduce noise and increase colony signal.

# Supplemental references For Supplemental Figure 2

Piatkevich, K. D., Hulit, J., Subach, O. M., Wu, B., Abdulla, A., Segall, J. E., et al. (2010). Monomeric red fluorescent proteins with a large Stokes shift. *Proc. Natl. Acad. Sci. U. S. A.* 107, 5369–5374. doi:10.1073/pnas.0914365107.

Shcherbakova, D. M., Hink, M. A., Joosen, L., Gadella, T. W. J., and Verkhusha, V. V. (2012). An orange fluorescent protein with a large stokes shift for single-excitation multicolor FCCS and FRET imaging. *J. Am. Chem. Soc.* 134, 7913–7923. doi:10.1021/ja3018972.

Shen, Y., Chen, Y., Wu, J., Shaner, N. C., and Campbell, R. E. (2017). Engineering of mCherry variants with long Stokes shift, red-shifted fluorescence, and low cytotoxicity. *PLoS One* 12, 1–14. doi:10.1371/journal.pone.0171257.
