# Supplementary material for: Teaching an old ‘doc’ new tricks for algal biotechnology: Strategic filter use enables multi-scale fluorescent protein signal detection
Source: Front Bioeng Biotechnol. 2022 Sep 23;10:979607. doi: 10.3389/fbioe.2022.979607 (PMC9540369; doi:10.3389/fbioe.2022.979607)
Supplement: Supplementary file 3 [file DataSheet3.PDF]

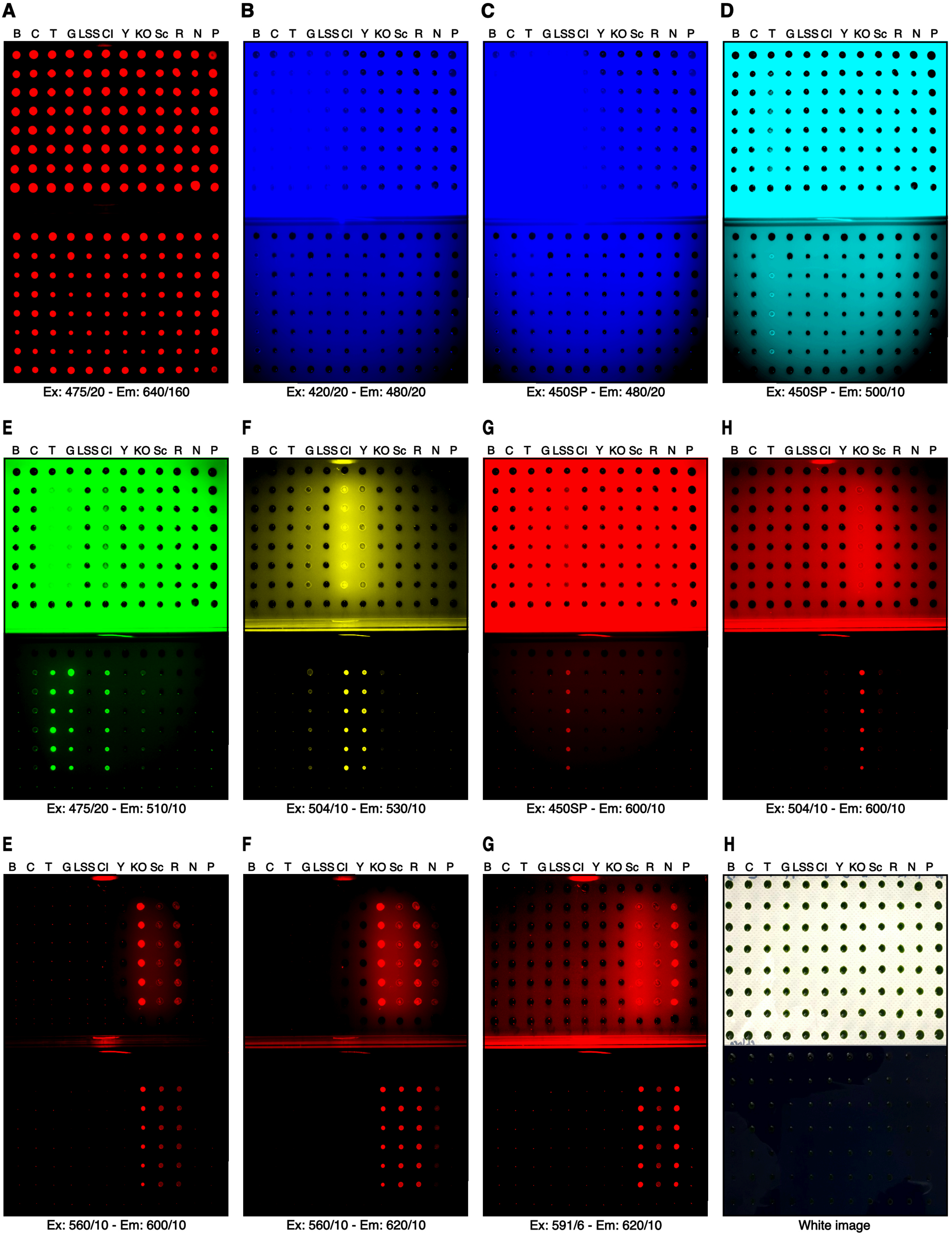

Supplemental Figure 1

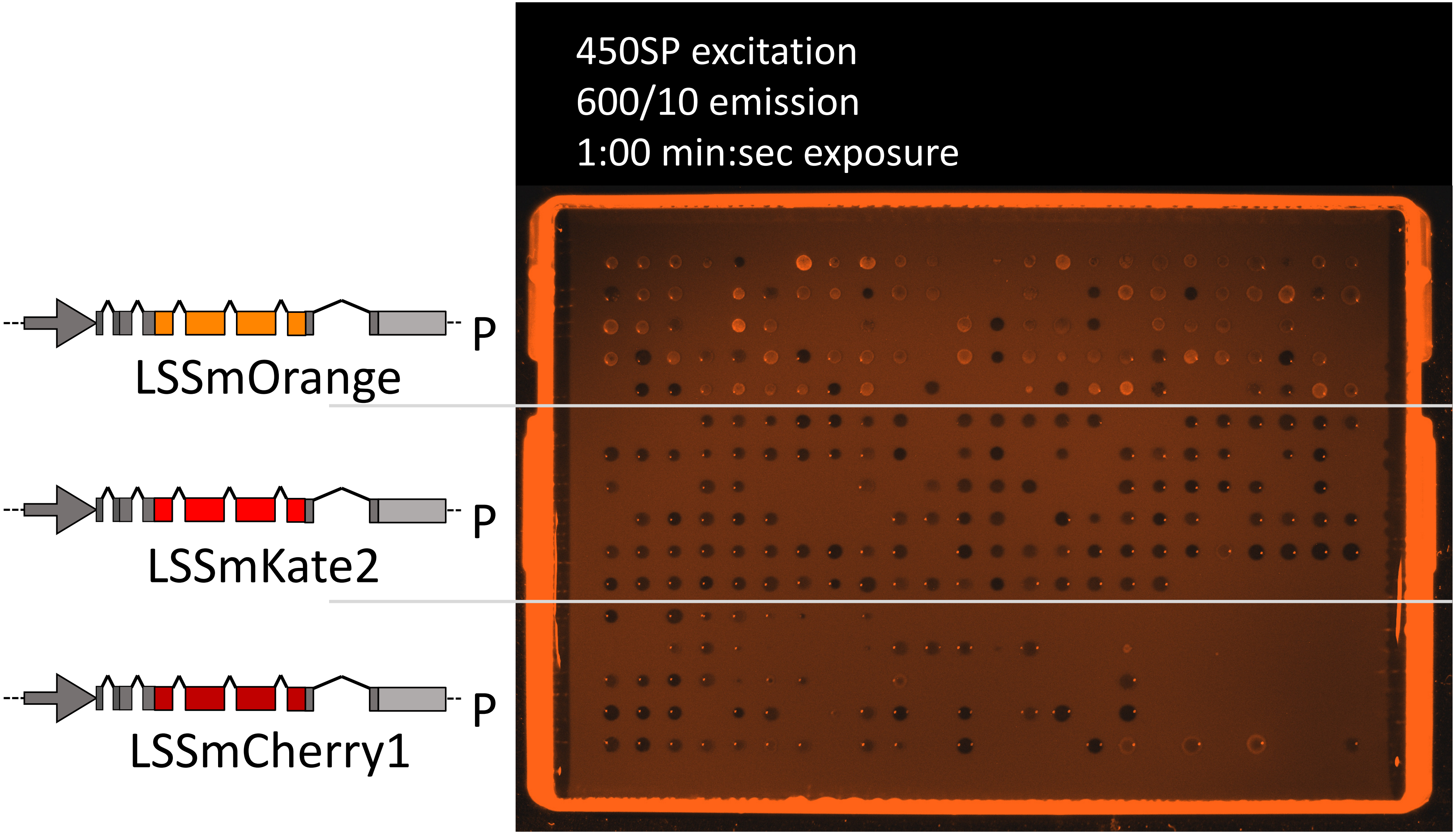

Protein name	Excitation:Emission λ max	Reference
LSSmKate2	460:605	(Piatkevich et al., 2010)
LSSmCherry1	450:610	(Shen et al., 2017)
LSSmOrange	437:572	(Shcherbakova et al., 2012)

Supplemental Figure 2

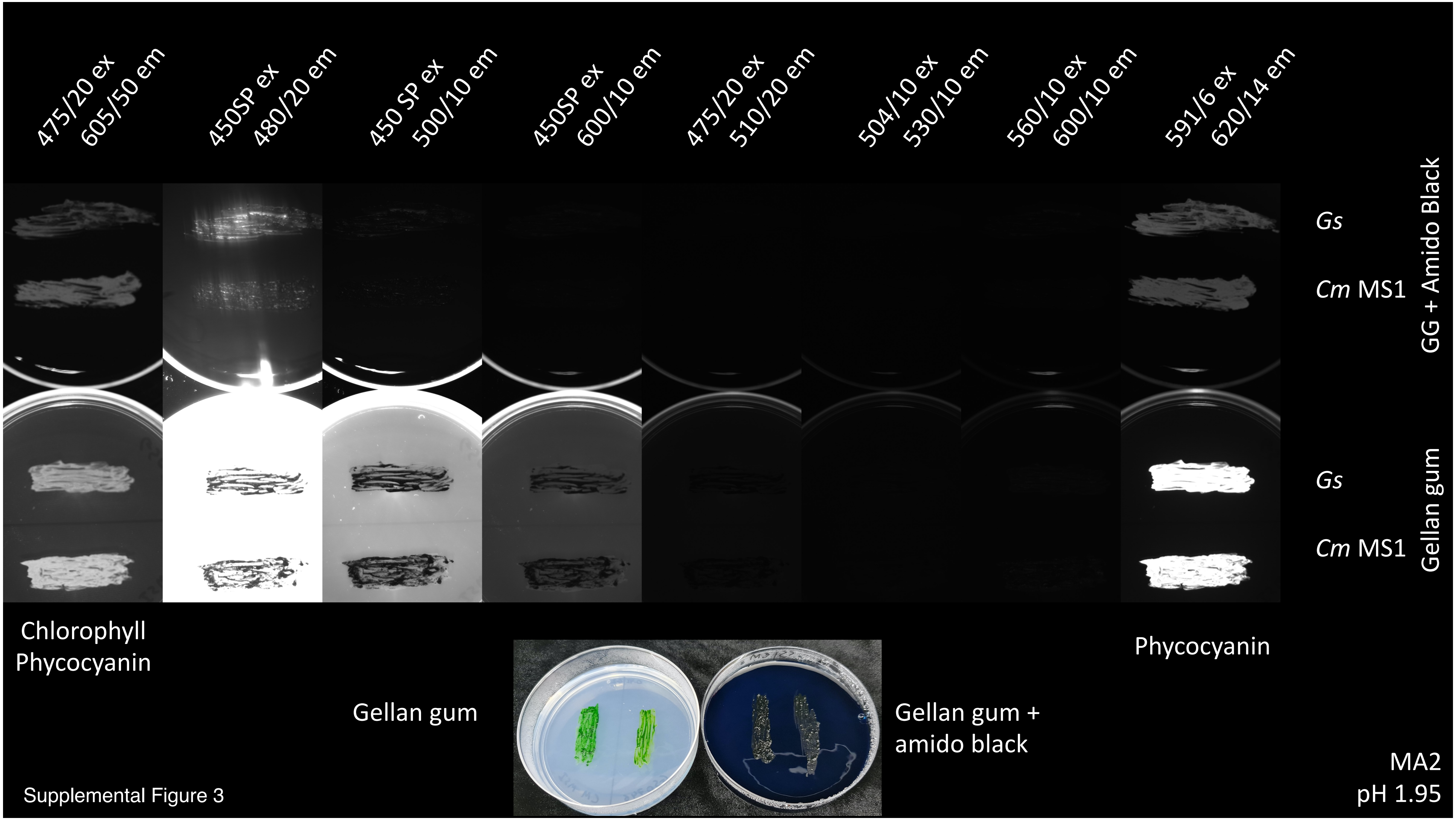

Supplemental Figure 3

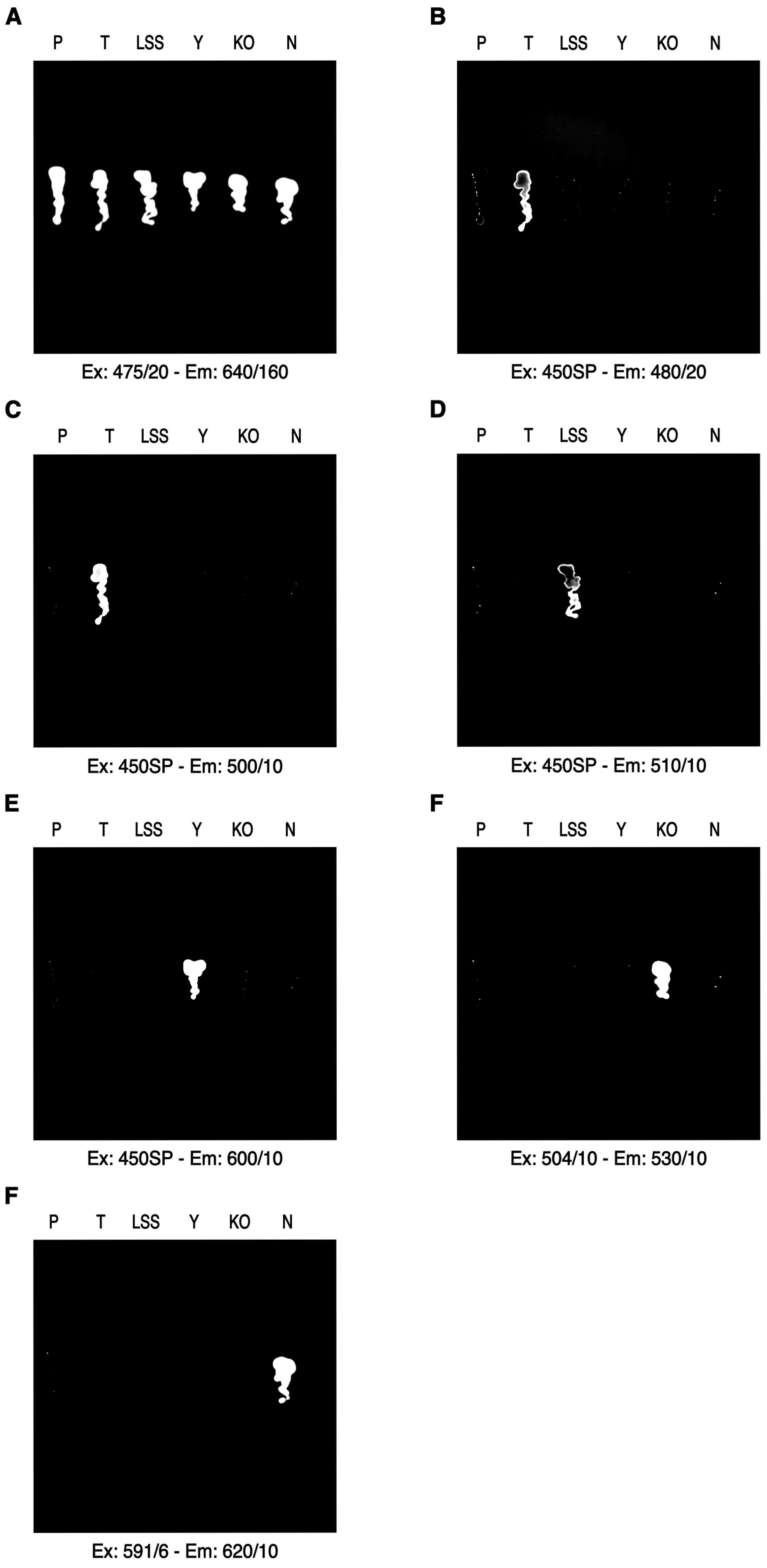

Supplemental Figure 4

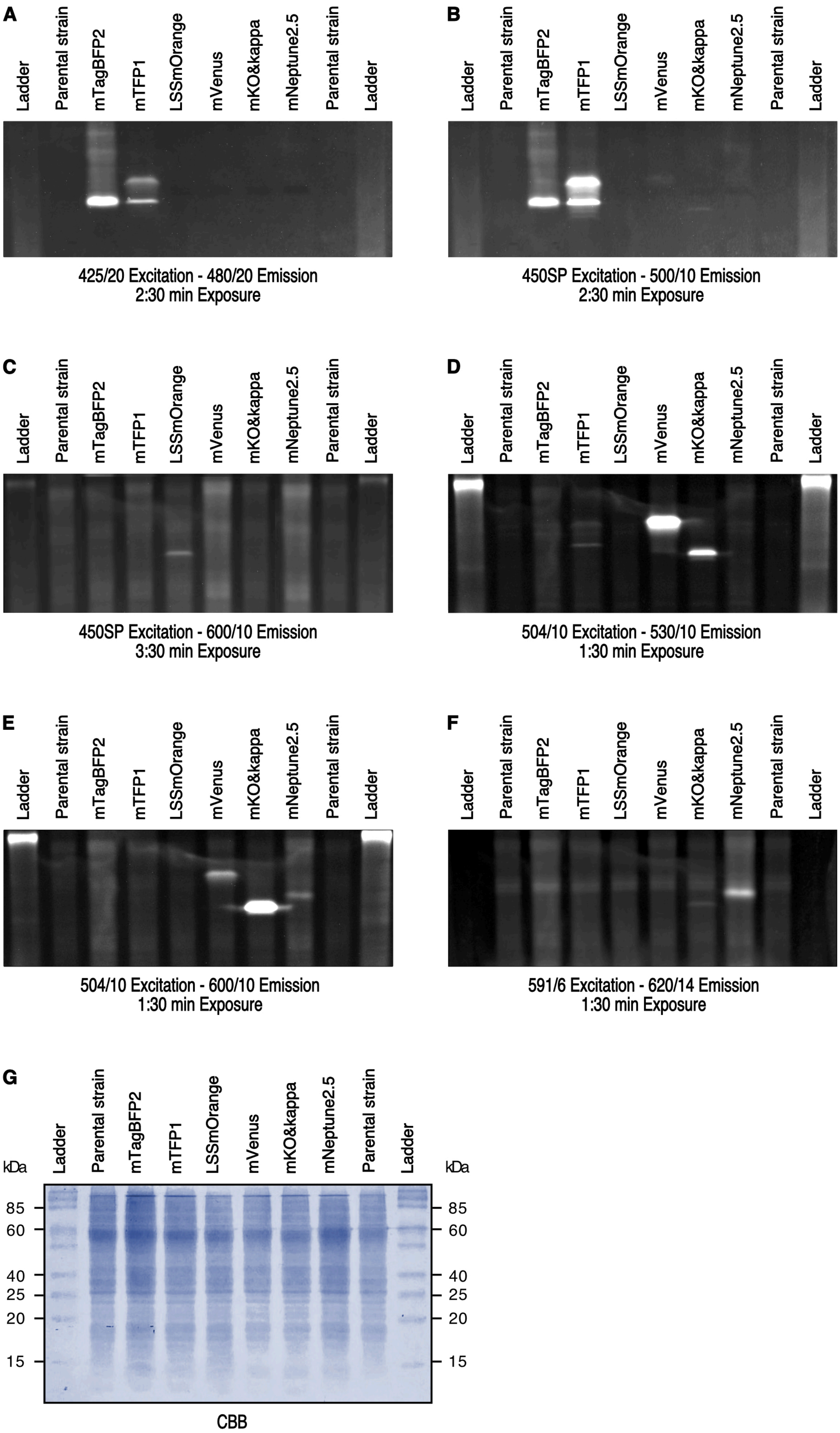

Supplemental Figure 5

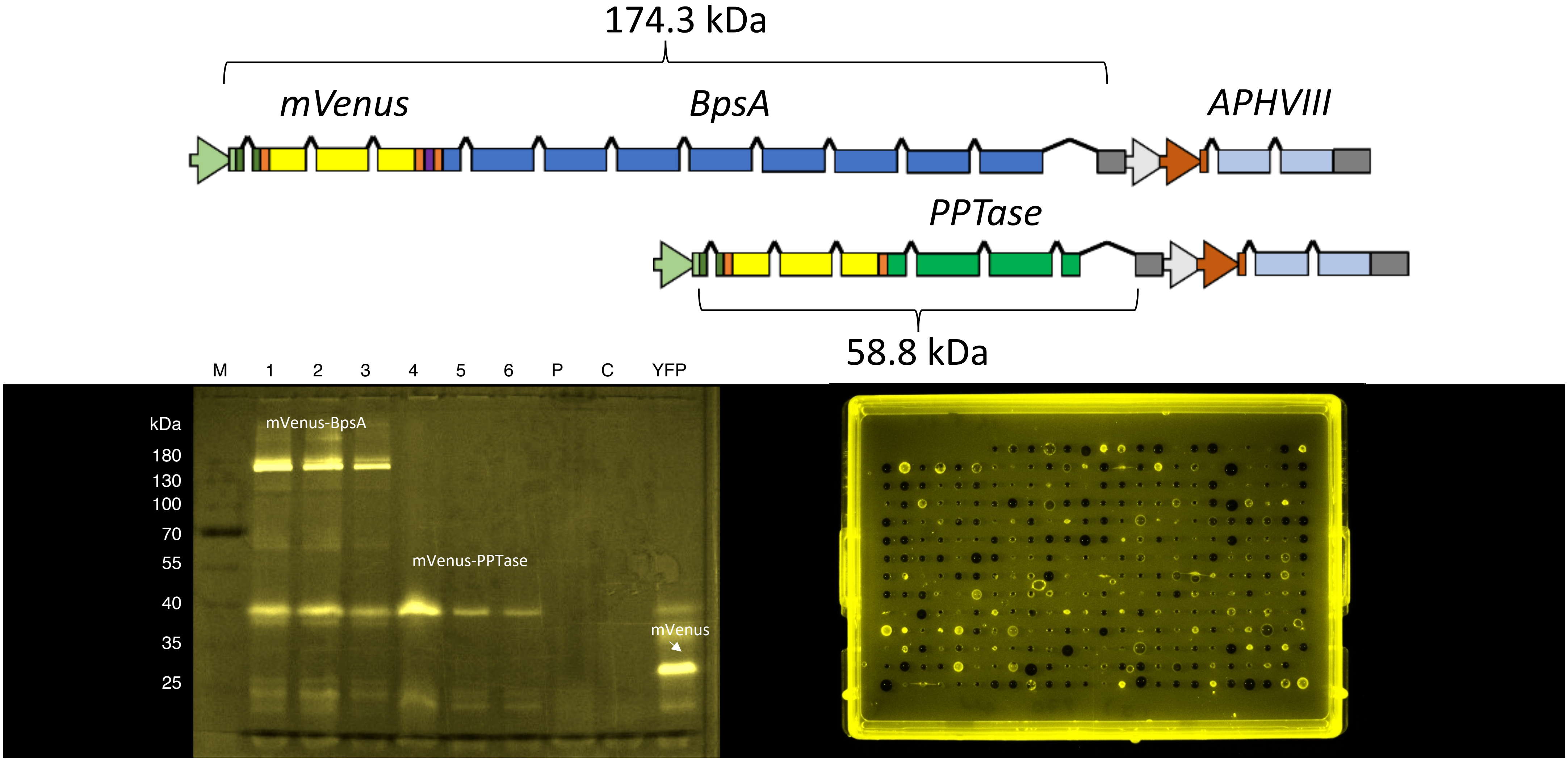

Supplemental Figure 6

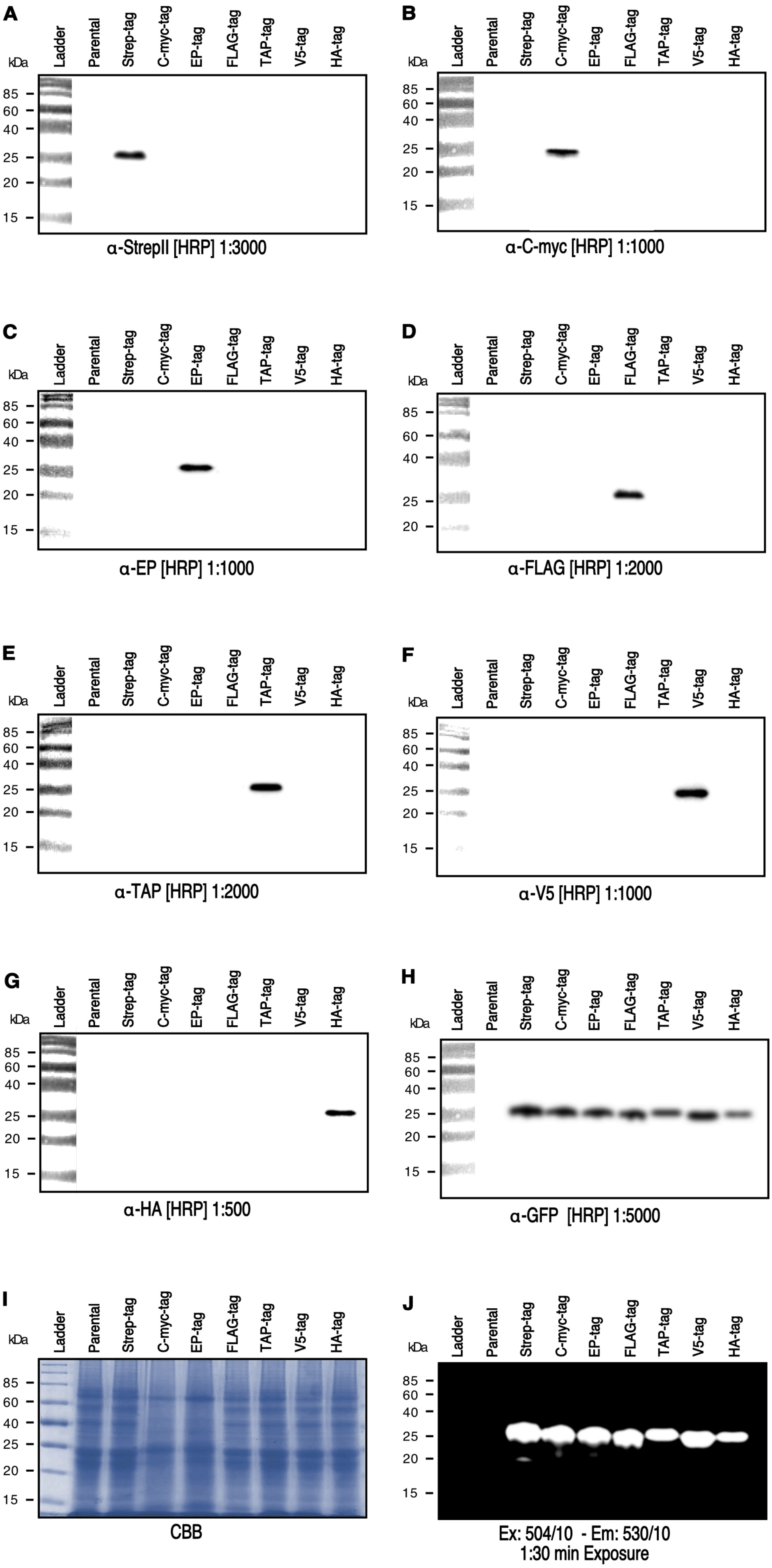

Supplemental Figure 7
